# Superior Overall Survival in Patients with Colorectal Cancer, Regular Aspirin Use, and Combined Wild-Type PIK3CA and KRAS-Mutated Tumors

**DOI:** 10.3390/cancers13194959

**Published:** 2021-10-01

**Authors:** Leonie Gebauer, Andrea Nist, Marco Mernberger, Thorsten Stiewe, Roland Moll, Kathleen Stabla, Uwe Klinge, Elisabeth Mack, Cornelia Brendel, Andreas Neubauer

**Affiliations:** 1 Clinic for Hematology, Oncology, Immunology, Center for Tumor Biology and Immunology, Philipps University of Marburg, Baldingerstrasse, 35037 Marburg, Germany; stablak@staff.uni-marburg.de (K.S.); elisabeth.mack@staff.uni-marburg.de (E.M.); brendelc@staff.uni-marburg.de (C.B.); neubauer@staff.uni-marburg.de (A.N.); 2 Department of Medicine III, University Hospital, LMU Munich, 81377 Munich, Germany; 3 Genomics Core Facility, Philipps-University of Marburg, 35043 Marburg, Germany; andrea.nist@imt.uni-marburg.de (A.N.); stiewe@uni-marburg.de (T.S.); 4 German Center for Lung Research (DZL), Institute of Molecular Oncology, Philipps University Marburg, 35043 Marburg, Germany; marco.mernberger@uni-marburg.de; 5 Institute of Pathology, University Hospital Giessen and Marburg GmbH, Philipps University of Marburg, Baldingerstrasse, 35043 Marburg, Germany; mollr@med.uni-marburg.de; 6 Department of General, Visceral, Transplant Surgery, University Hospital of the RWTH, 52074 Aachen, Germany; uklinge@ukaachen.de

**Keywords:** colorectal cancer, aspirin use, *PIK3CA*, *KRAS*

## Abstract

**Simple Summary:**

The impact of aspirin use after the diagnosis of colorectal cancer is unknown. Among others, *PIK3CA* mutational status was proposed as a molecular biomarker for the response to adjuvant aspirin therapy. The aim of this study was to retrospectively analyze whether the *PIK3CA* and *KRAS* mutational status had an impact on overall survival in patients with colorectal cancer and aspirin use. In a retrospective study, we obtained *KRAS* and *PIK3CA* mutational status in a cohort of 153 patients with a first diagnosis of colorectal cancer receiving tumor surgery with curative intent. Clinicopathological data and survival data were assessed using patient records and reporting registers. We observed a significant 10-year overall survival benefit in patients with aspirin use and combined wild-type *PIK3CA* and mutated-*KRAS* tumors (HR = 0.38; 95% CI = 0.17–0.87; *p* = 0.02). Our data indicated a benefit of aspirin usage particularly for patients with combined wild-type *PIK3CA* and mutated-*KRAS* tumor characteristics.

**Abstract:**

The impact of aspirin use after the diagnosis of colorectal cancer is unknown. Among others, *PIK3CA* (phosphatidylinositol-4,5-bisphosphate 3-kinase, catalytic subunit alpha) mutational status was proposed as a molecular biomarker for the response to adjuvant aspirin therapy. However, prognostic data on aspirin use after a colorectal cancer diagnosis in relation to *KRAS* mutational status is limited. In a single-center retrospective study, we obtained *KRAS* and *PIK3CA* mutational status in a cohort of 153 patients with a first diagnosis of colorectal cancer receiving tumor surgery with curative intent. *PIK3CA* mutational status was determined by pyrosequencing, and *KRAS* mutational status was determined by next-generation sequencing. Clinicopathological data and survival data were assessed using patient records and reporting registers. We observed a significant 10-year overall survival benefit in patients with aspirin use and combined wild-type *PIK3CA* and mutated-*KRAS* tumors (HR = 0.38; 95% CI = 0.17–0.87; *p* = 0.02), but not in patients without aspirin use. Our data indicate a benefit of aspirin usage particularly for patients with combined wild-type *PIK3CA* and mutated-*KRAS* tumor characteristics.

## 1. Introduction

With 1.4 million new cases per year, colorectal cancer is one of the most common cancers worldwide. Moreover, colorectal cancer is one of the most common causes of cancer-related death, with almost 700,000 deaths per year [1]. Therefore, it is of interest to understand the molecular mechanisms of cancer development and metastasis to identify new therapeutic approaches.

Retrospective analyses suggest a superior clinical outcome for patients with regular use of aspirin after diagnosis of colorectal cancer [2,3,4,5,6,7]. Aspirin is a commonly prescribed drug in Western societies, and its indication includes prevention and therapy of coronary artery disease and stroke [8,9]. Nevertheless, the long-term use of aspirin may provoke side effects such as intracranial and gastrointestinal bleeding, stomach ulcers, and renal impairment [10]. Therefore, it is highly desirable to identify those colorectal cancer patients with an expectable overweight of benefits [11,12]. Several possible biomarkers have been suggested, including the *PIK3CA* (phosphatidylinositol-4,5-bisphosphate 3-kinase, catalytic subunit alpha) mutational status [13,14,15,16,17]. Some studies indicated a favorable survival or longer recurrence-free survival of aspirin users with mutated-*PIK3CA* tumors [13,14], while others reported increased survival of aspirin users with wild-type *PIK3CA* tumors [17]. Two other independent studies did not point to survival differences with regard to *PIK3CA* mutations and aspirin use [15,16]. Prognostic data on aspirin use after diagnosis of colorectal cancer in relation to *KRAS* mutational status is limited.

The mechanisms by which aspirin could prolong survival in patients with colorectal cancer have not been clarified. The prognosis-improving effect in various solid tumors appears to be mediated on the one hand by inhibition of cyclooxygenase, and on the other hand via cyclooxygenase-independent signaling pathways [18,19]. In animal models, it has been shown that inhibitors of cyclooxygenase hamper both tumor growth and metastasis in colorectal cancers [20,21]. In addition, it has been demonstrated experimentally that inhibition of cyclooxygenase-2, which is overexpressed in a large number of colorectal cancers, leads to reduced production of prostaglandin E2 (PGE2), which favors cell migration, angiogenesis, and reduced apoptosis [22,23,24].

The aim of this work was to examine whether *PIK3CA* mutational status and *KRAS* mutational status could indeed augment the effect of aspirin on the survival of patients with colorectal cancer.

## 2. Materials and Methods

### 2.1. Patient Cohort

**Figure 1:**
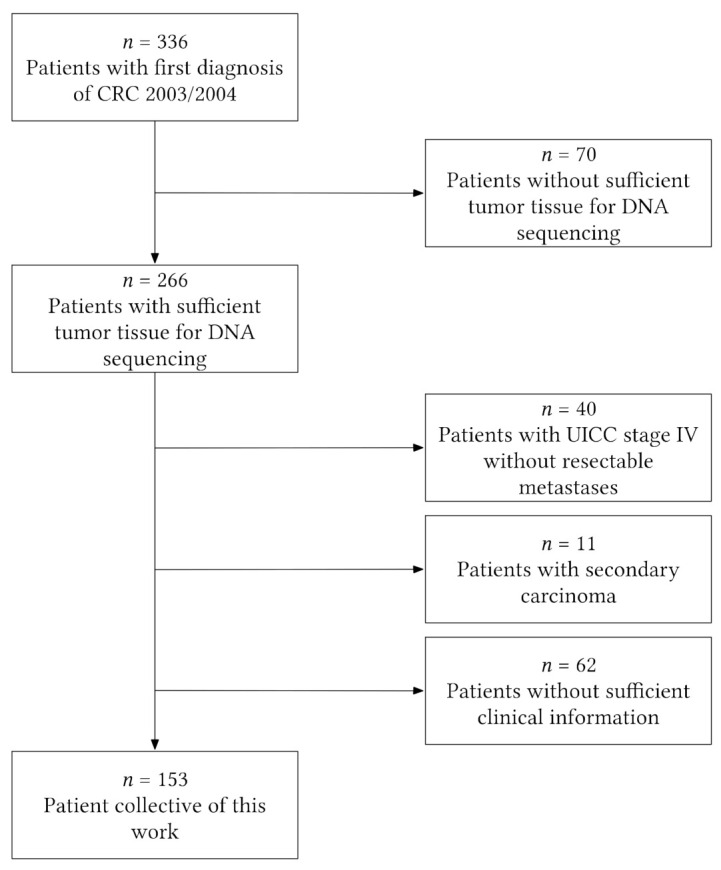
Flowchart illustrating patient-exclusion criteria leading to the final study cohort of 153 patients. CRC = colorectal cancer.

We performed a retrospective single-center analysis at the university clinic in Marburg, Germany. Therefore, we used a database consisting of 336 patients with histologically proven diagnoses of sporadic colorectal cancer that underwent curative surgical resection at the university clinic of Marburg in 2003/2004. For 266 of those 336 patients, tissue for DNA analysis was available; 113 of these patients had to be excluded because they had been diagnosed with stage IV (UICC classification) with no resectable metastases, had a secondary tumor, or did not have sufficient clinical information (information on aspirin intake/survival data). Finally, the study cohort consisted of 153 patients (Figure 1).

Information on aspirin use was obtained from the case files or the primary care physician; and information on survival data from the case file, primary care physician, or register of residence. Aspirin use was defined as regular use of low-dose aspirin (100–300 mg) during most weeks after a diagnosis of colorectal cancer, whereas nonuse of aspirin was defined as no regular use of aspirin during most weeks. The indications for aspirin use included primary or secondary prevention of cardiovascular disease.

Paraffin-embedded tissue blocks were provided by the pathology archive of the Institute of Pathology of the university clinic of Marburg. The study was performed according to the guidelines of the local ethics committee (no. 98/20).

### 2.2. Detection of PIK3CA Mutations

Genomic DNA was isolated from paraffin-embedded tumor tissue samples (QIAamp DNA Mini Kit, Germantown, MD, USA), and DNA amplification was performed by polymerase chain reaction (PCR) with primers from Nosho et al. [25]. Point mutations in exon 9 and 20 of *PIK3CA* were analyzed by pyrosequencing. The PCR was performed in a 50 µL volume containing 4 µL of template DNA (50 ng/µL), 1×PCR-Buffer, MgCl2, 10 mM (each 2.5 mM) deoxynucleoside triphosphate (dNTP), 10 µM forward-biotin primer, 10 µM reverse primer, and 1.25 U Platinum Taq DNA polymerase (Life Technologies, Carlsbad, CA, USA). PCR conditions were as follows: 95 °C, 11 min; 45 cycles of 95 °C, 30 s; 54 °C, 30 s; 72 °C, 30 s; then 72 °C, 5 min. PCR products were sequenced by pyrosequencing (PyroMark^®^ Q24, Qiagen, Germantown, MD, USA) using the Therascreen^®^ kit (Qiagen, Germantown, MD, USA) according to the manufacturer’s instructions with primers from Nosho et al. [25]. The different pyrosequencing primers for *PIK3CA* exon 9 were 9-RS1, 5′-ccatagaaaatctttctcct-3′ (mutations: c.1634A>G, c.1636C>A); 9-RS2, 5′-tagaaaatctttctcctgct-3′ (mutations: c.1633G>A, c.1624G>A)′; 9-RS3, 5′-ttctccttgcttcagtgattt-3′ (mutation c.1624G>A); and for *PIK3CA* exon 20: 20-RS, 5′-gttgtccagccacca-3′ (mutations:c.3140A>G, c.3140A>T, c.3129G>T, c.3139C>T).

### 2.3. Detection of KRAS Mutations

The human *KRAS* locus corresponding to codons 4 to 16 was amplified from genomic DNA isolated from tumor microdissections. Each sample was tagged with a unique 8-nucleotide barcode combination using 12 different forward (5′-AAT[barcode] TTATAAGGCCTGCTGAAAATGACTGAA-3′) and eight different reverse (5′-AAT[barcode]TGAATTAGCTGTATCGTCAAGGCACT-3′) primers per 96-well plate. PCR products from a single 96-well plate were pooled and purified, and an indexed sequencing library was prepared using the IonXpress Plus Fragment Library Prep Kit in combination with the IonXpress Barcode Adapters 1–16 Kit (Life Technologies, Carlsbad, CA, USA). The quality of sequencing libraries was verified on a Bioanalyzer DNA High Sensitivity chip (Agilent, Santa Clara, CA, USA); subsequently, libraries were pooled and quantified by qPCR. Sequencing was carried out on the IonTorrent PGM (Life Technologies, Carlsbad, CA, USA) according to the manufacturer’s recommendations. After demultiplexing and removal of the tag sequences using Cutadapt v1.7 (TU Dortmund, Dortmund, DE, Germany), reads were aligned to Ensembl v70 (European Bioinformatics Institute, Hinxton, UK) using Bowtie 2 (version 2.0.0-beta7, JHU, Baltimore, MD, USA). Variants in the *KRAS* coding region were obtained using VarScan 2 (version 2.3.5, WUSTL, St. Louis, MO, USA), using a minimum variant frequency of 2.5% and minimum read coverage of 2000.

### 2.4. Statistical Analysis

Statistical analysis was performed using Statistical Package for Social Sciences software (SPSS^®^ v23 (IBM, Armonk, NY, USA)) and Prism Software version 6.0 (GraphPad Software, San Diego, CA, USA). Age (years), sex, tumor location, histological grade, T-size, lymph nodes, metastasis, UICC stage, *PIK3CA* status, *KRAS* status, survival at 5 years, survival at 10 years, survival time (days), and presence of two mutations were checked for significant differences between ASS users and ASS nonusers by use of a Mann–Whitney test. Fisher’s exact test and a logrank (Mantel-Cox) test were used to test for differences in overall survival; *p* < 0.05 was considered statistically significant.

## 3. Results

### 3.1. Patients

The study group consisted of 153 patients with histologically proven diagnosis of colorectal cancer. The follow-up of the study group was until death or up to 120 months. Table 1 summarizes the baseline characteristics of the patients with colorectal cancer according to aspirin use or nonuse after diagnosis, and the presence or absence of tumor *PIK3CA* mutation.

Mutations in the *PIK3CA* gene were found in 15.7% of all patients. Of all mutations in the *PIK3CA* gene, there were 6 mutations in exon 20 and 18 mutations in exon 9. There were no double mutations (exon 9 and exon 20). The most common mutation was the c.1633G>A mutation in exon 9, followed by the c.1624G>A mutation in exon 9 and the c.3140A>G mutation in exon 20. Mutations in the *KRAS* gene were found in 56.9% of all patients. The most common mutation was the glycine/aspartate substitution in codon 13 (p.G13D, 31%), followed by the glycine/aspartate substitution in codon 12 (p.G12D, 26%). Aspirin was taken by 34% of the patients in daily dosages of 100–300 mg. The 10-year overall survival of the entire study cohort was 43.8%.

Tumor mutations in the *KRAS* gene were equally distributed between patients with wild-type *PIK3CA* tumors and patients with mutated-*PIK3CA* tumors: An additional *KRAS* mutation was found in 13/24 (54%) of the mutated-*PIK3CA* tumors and in 74/129 (57%) of the wild-type *PIK3CA* tumors (Fisher’s exact test, *p* = 0.82). A total of 34% of the patients with wild-type *PIK3CA* tumors and 33% of the patients with a tumor mutation in the *PIK3CA* gene took aspirin regularly after the diagnosis of colorectal cancer.

The subgroups with mutated-*PIK3CA* tumors and wild-type *PIK3CA* tumors and the subgroups with mutated-*KRAS* tumors and wild-type *KRAS* tumors were similar according to the baseline characteristics (mean age at diagnosis, sex, tumor location, stage of disease, tumor grading, and aspirin use before diagnosis; *p* > 0.1 for all comparisons).

Moreover, the subgroups with and without aspirin use were similar according to the baseline characteristics (mean age at diagnosis, sex, tumor location, stage of disease, and mutational status; *p* > 0.1 for all comparisons).

### 3.2. Aspirin Use and Survival in the Cohort of 153 Patients

**Figure 2:**
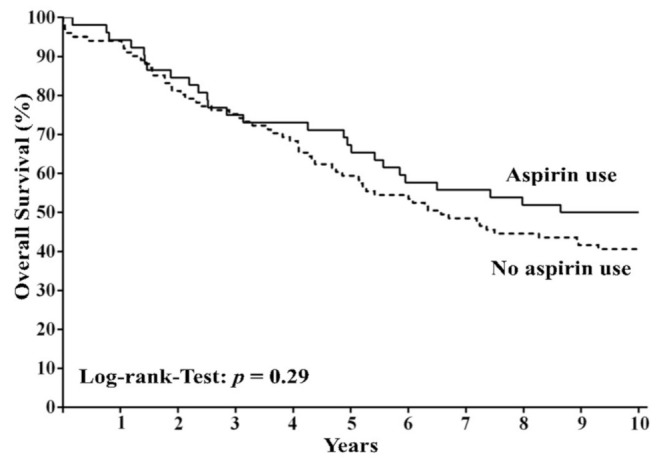
Overall survival among patients with colorectal cancer according to regular use or nonuse of aspirin after diagnosis. The panel shows overall survival among patients with regular use of aspirin and those with nonuse of aspirin.

With regard to all 153 patients, patients with regular use of aspirin (*n* = 52) did not show significantly improved 10-year overall survival compared to patients without regular use of aspirin (*n* = 101) (HR = 0.77; 95% CI = 0.5–1.22; *p* = 0.29; Figure 2).

### 3.3. Aspirin Use and Survival According to PIK3CA und KRAS Mutational Status

**Figure 3:**
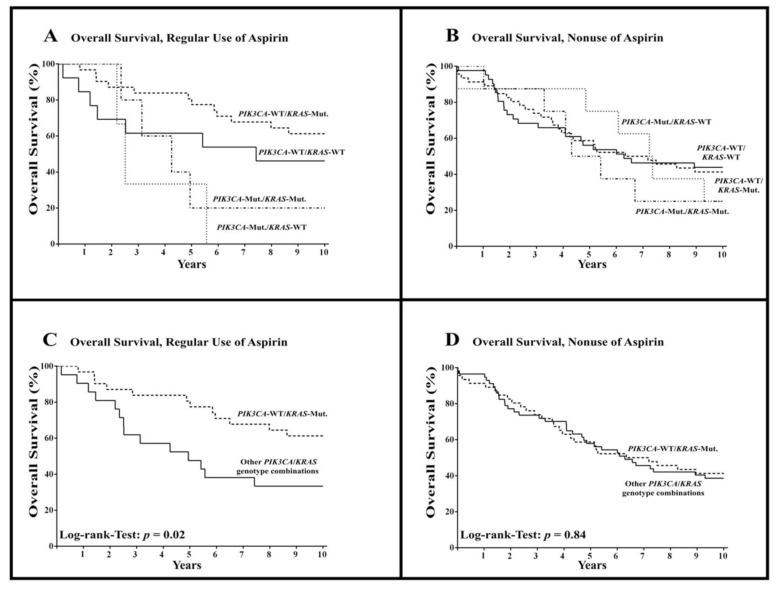
Overall survival among patients with colorectal cancer according to *PIK3CA* and *KRAS* mutational status and regular use or nonuse of aspirin after diagnosis. WT = wild-type, Mut. = mutated. (**A**,**B**) show overall survival among all combinations of mutational status in patients with regular use of aspirin (**A**) and nonuse of aspirin (**B**), respectively, while (**C**,**D**) show overall survival among patients with combined wild-type-*PIK3CA* and mutated-*KRAS* tumors compared to other *PIK3CA/KRAS* genotype combinations (except *PIK3CA-WT/KRAS*-Mut.).

Among all patients with regular use of aspirin, we found patients with wild-type *PIK3CA* tumors to harbor a significant overall survival advantage as compared to patients with mutated-*PIK3CA* tumors (HR = 0.33; 95% CI = 0.05–0.62; *p* < 0.01). This survival advantage was not evident among the patients without aspirin use (HR = 0.78; 95% CI = 0.38–1.51; *p* = 0.43). A combined examination of the mutational status in the *KRAS* and *PIK3CA* genes revealed clear differences in the absolute 10-year survival rates among patients who regularly used aspirin (Figure 3A) but less differences among patients without regular aspirin use (Figure 3B).

When reviewing the combined *PIK3CA* and *KRAS* mutational status of patients with regular aspirin use, a significant overall survival benefit for patients with combined wild-type *PIK3CA* and mutated-*KRAS* tumors was observed (HR = 0.38; 95% CI = 0.17–0.87; *p* = 0.02; Figure 3C, Table 2). This survival advantage was not observed in patients without aspirin use (HR = 0.95; 95% CI = 0.58–1.57; *p* = 0.84; Figure 3D, Table 2).

## 4. Discussion

We found that patients with regular aspirin use after the diagnosis of colorectal cancer and combined wild-type *PIK3CA* and mutated-*KRAS* tumors showed a significant survival benefit. Several studies have investigated a potential correlation between *PIK3CA* mutational status and benefit from postdiagnosis aspirin use [13,14,15,16,17]. Some studies have indicated a better survival or longer recurrence-free survival of aspirin users with mutated-*PIK3CA* tumors [13,14], while others found a favorable survival of aspirin users with wild-type *PIK3CA* tumors [17]. Two other studies did not point to survival differences with regard to *PIK3CA* mutational status and aspirin use [15,16]. To the best of our knowledge, our study is the first report that describes a positive effect of aspirin usage in a cohort of colon cancer patients in the context of both *PIK3CA*- and *KRAS*-mutational status.

Colorectal cancer is a heterogeneous group of diseases, and it can be assumed that combinations of prognostic factors and not individual biomarkers alone influence the response to therapy, such as an “adjuvant” therapy with aspirin [11,12]. A possible explanation for the divergent results of the studies [13,14,15,16,17] concerning the *PIK3CA* mutational status, aspirin use, and overall survival could be that the patient cohorts of the studies differed with regard to further molecular tumor markers. When comparing the patient collective of this work with the patient collectives of similar works, differences in tumor location and *KRAS* mutational status were found [13,14,15,16,17]. This could indicate that patient collectives differed in terms of molecular tumor properties. In this study, 25% of the patients had a right-sided colorectal cancer and 57% showed *KRAS* mutations, whereas the proportion of patients with a right-sided colorectal cancer in the study of Liao et al. [13] was significantly higher (45%), and the number of *KRAS* mutations was significantly lower (35%) [13]. Left-sided colorectal cancers as compared to right-sided colorectal cancers show a different distribution of mutations and harbor a divergent prognosis [26]. For example, the incidence of microsatellite instability and mutations in the *BRAF* gene is increasing from the rectum to the proximal colon [26,27]. The patient cohorts in the different papers could therefore differ with regard to the molecular background. Unfortunately, based on the available data, it was not possible to compare this.

A possible alternative explanation for our findings could be that there are other biomarkers that predict response to aspirin and correlate with the *KRAS* or *PIK3CA* mutational status. Large prospective randomized studies are required to evaluate the response to “adjuvant” therapy with aspirin in colorectal cancer by considering various combinations of possible biomarkers.

Furthermore, it is important to understand the underlying molecular mechanism of how aspirin could improve the prognosis of colorectal cancer to detect relevant biomarkers. It has recently been shown that the interaction between *KRAS* and the *PIK3CA* catalytic subunit p110α decreases significantly after the addition of aspirin, and leads to an inhibition of cell proliferation and a cell-cycle arrest in the G0/G1 phase [28]. Thus, aspirin may inhibit cell growth of leiomyoma cells by regulating the K-Ras-p110α interaction. In particular, cells with overexpressed *KRAS* displayed an increased K-Ras-p110α interaction [28].

It is not clear whether these molecular mechanisms also play a role in colorectal cancer cells. Due to the inhibition of the K-Ras-p110α interaction by aspirin, it would be plausible that especially patients with simultaneous occurrence of mutated-*KRAS* and wild-type *PIK3CA* tumors could benefit from aspirin use. Further studies are required to determine if these signaling pathways play a role in patients with colorectal cancer in the context of aspirin use.

Besides signal transduction changes induced by aspirin, autophagy may also be modulated [29,30,31,32,33]. Whether changes in autophagy or its signal modulation via the classical inhibition of COX (cyclooxygenase) or even ERK (extracellular signal-regulated kinase) was causative for the described effect needs to be addressed in prospective clinical trials.

Of note, although the total number of analyzed patients was rather small, the benefit of aspirin use in patients with combined wild-type *PIK3CA* and mutated-*KRAS* tumors was clearly denoted. The frequency of *PIK3CA* mutations in our study fit well with the data presented by others, and the clinical and pathological data matched very well with the published data [13,14,15,16,17,34,35]. Nevertheless, because of the small numbers of patients in some subgroups, we must be cautious in interpreting our data. However, our data call for larger randomized trials, especially since some of some of the trials included only patients with mutated-*PIK3CA* tumors.

This was a unicentric German study, and thus may be biased by single-center experience. Recent data indicated a distinct treatment response to chemotherapy particular in German versus non-German patients with rectal adenocarcinoma [36]. Our data from an unicenter German patient cohort may therefore not be representative for patients from other countries, and warrant further exploration in a prospective study.

Taken together, these data suggest that the shared consideration of both the *PIK3CA* and *KRAS* mutational status could play an important role. These data must be confirmed in larger prospective clinical trials. This is especially critical, as some clinical trials recruit colorectal cancer patients into aspirin trials only in case the tumors harbor *PIK3CA* mutations (see: clinicaltrials.gov, NCT02467582).

## 5. Conclusions

In summary, the results of this work suggest that patients with a combination of wild-type *PIK3CA* and mutated-*KRAS* tumors in particular benefit from aspirin use after diagnosis of colorectal cancer. The results of this study conclusively underline that the roles of the *PIK3CA* and *KRAS* mutational status as potential predictive markers for adjuvant therapy with aspirin in patients with colorectal cancer require prospective studies.

## Figures and Tables

**Table 1:** Baseline characteristics of patients with colorectal cancer, according to *PIK3CA* (phosphatidylinositol-4,5-bisphosphate 3-kinase, catalytic subunit alpha) mutational status and regular use or nonuse of aspirin after diagnosis.

Characteristics	All Patients (*n* = 153)	Wild-Type *PIK3CA*		Mutant *PIK3CA*	
		No Aspirin Use	Aspirin Use	No Aspirin Use	Aspirin Use
Sex—no. (%)					
Male	90 (59%)	46 (54%)	26 (59%)	11 (69%)	7 (88%)
Female	63 (41%)	39 (46%)	18 (41%)	5 (31%)	1 (12%)
Age—year	69.9 ± 7.7	68.8 ± 11.6	71.6 ± 7.6	69.4 ± 9.0	72.4 ± 5.0
Tumor location—no. (%)					
Rectum	46 (30%)	25 (29%)	17 (39%)	3 (19%)	1 (13%)
Distal colon	68 (44%)	42 (49%)	15 (34%)	6 (38%)	5 (63%)
Proximal colon	39 (25%)	18 (21%)	12 (27%)	7 (44%)	2 (25%)
Disease stage—no. (%)					
I	39 (25%)	22 (26%)	10 (23%)	4 (25%)	3 (37%)
II	53 (35%)	31 (36%)	16 (36%)	5 (31%)	1 (13%)
III	56 (37%)	29 (34%)	16 (36%)	7 (44%)	4 (50%)
IV	5 (3%)	3 (4%)	2 (5%)	0 (0%)	0 (0%)
Tumor differentiation—no. (%)					
G1	5 (3%)	2 (2%)	1 (2%)	0 (0%)	2 (25%)
G2	133 (87%)	73 (86%)	41 (93%)	15 (94%)	4 (50%)
G3	15 (10%)	10 (12%)	2 (5%)	1 (6%)	2 (25%)
*KRAS*					
Wild-type	66 (43%)	41 (48%)	14 (32%)	8 (50%)	3 (38%)
Mutant	87 (57%)	44 (52%)	30 (68%)	8 (50%)	5 (62%)

**Table 2:** The 10-year overall survival among patients with regular use or nonuse of aspirin after diagnosis of colorectal cancer according to *PIK3CA* and *KRAS* mutational status.

Aspirin Use	Number of Patients	Dead at 10 Years after Diagnosis	Alive at 10 Years after Diagnosis	*p*-Value
Yes	0.02			
*PIK3CA-WT/KRAS*-Mut.	31	12 (39%)	19 (61%)	
Other *PIK3CA/KRAS* genotype combinations (except *PIK3CA-WT/KRAS*-Mut.)	21	14 (67%)	7 (33%)	
No	0.84			
*PIK3CA-WT/KRAS*-Mut.	45	26 (58%)	19 (42%)	
Other *PIK3CA/KRAS* genotype combinations (except *PIK3CA-WT/KRAS*-Mut.)	56	34 (61%)	22 (39%)	

The *p*-values were calculated with the use of a logrank test.

## Data Availability

The data presented in this study can be made available upon request from the corresponding author.

## References

[1] Stewart B.W., Wild C.P. (2014). World Cancer Report 2014.

[2] Chan A.T., Ogino S., Fuchs C.S. (2009). Aspirin use and survival after diagnosis of colorectal cancer. Jama.

[3] Rothwell P.M., Wilson M., Elwin C.-E., Norrving B., Algra A., Warlow C.P., Meade T.W. (2010). Long-term effect of aspirin on colorectal cancer incidence and mortality: 20-year follow-up of five randomised trials. Lancet.

[4] Rothwell P.M., Wilson M., Price J.F., Belch J.F., Meade T.W., Mehta Z. (2012). Effect of daily aspirin on risk of cancer metastasis: A study of incident cancers during randomised controlled trials. Lancet.

[5] Bains S.J., Mahic M., Myklebust T.A., Smastuen M.C., Yaqub S., Dorum L.M., Bjornbeth B.A., Moller B., Brudvik K.W., Tasken K. (2016). Aspirin As Secondary Prevention in Patients with Colorectal Cancer: An Unselected Population-Based Study. J. Clin. Oncol..

[6] Goh C.H., Goh H.H., Leong W.Q., Chew M.H., Pan Y.S., Tony L.K.H., Chew L., Tan I.B.H., Toh H.C., Tang C.L. (2014). Post-operative aspirin use and colorectal cancer-specific survival in patients with stage I-III colorectal cancer. Anticancer Res..

[7] McCowan C., Munro A.J., Donnan P.T., Steele R.J.C. (2013). Use of aspirin post-diagnosis in a cohort of patients with colorectal cancer and its association with all-cause and colorectal cancer specific mortality. Eur. J. Cancer.

[8] Hennekens C.H., Schneider W.R. (2008). The need for wider and appropriate utilization of aspirin and statins in the treatment and prevention of cardiovascular disease. Expert Rev. Cardiovasc. Ther..

[9] Ittaman S.V., VanWormer J.J., Rezkalla S.H. (2014). The role of aspirin in the prevention of cardiovascular disease. Clin. Med. Res..

[10] Williams C.S., Mann M., DuBois R.N. (1999). The role of cyclooxygenases in inflammation, cancer, and development. Oncogene.

[11] Ogino S., Fuchs C.S., Giovannucci E. (2012). How many molecular subtypes? Implications of the unique tumor principle in personalized medicine. Expert Rev. Mol. Diagn..

[12] Coyle C., Cafferty F.H., Langley R.E. (2016). Aspirin and Colorectal Cancer Prevention and Treatment: Is It for Everyone?. Curr. Colorectal Cancer Rep..

[13] Liao X., Lochhead P., Nishihara R., Morikawa T., Kuchiba A., Yamauchi M., Imamura Y., Qian Z.R., Baba Y., Shima K. (2012). Aspirin use, tumor PIK3CA mutation, and colorectal-cancer survival. N. Engl. J. Med..

[14] Domingo E., Church D.N., Sieber O., Ramamoorthy R., Yanagisawa Y., Johnstone E., Davidson B., Kerr D.J., Tomlinson I.P.M., Midgley R. (2013). Evaluation of PIK3CA mutation as a predictor of benefit from nonsteroidal anti-inflammatory drug therapy in colorectal cancer. J. Clin. Oncol..

[15] Kothari N., Kim R., Jorissen R.N., Desai J., Tie J., Wong H.-L., Faragher I., Jones I., Day F.L., Li S. (2015). Impact of regular aspirin use on overall and cancer-specific survival in patients with colorectal cancer harboring a PIK3CA mutation. Acta Oncol..

[16] Murphy C., Turner N., Wong H.-L., Sinnathamby M., Tie J., Lee B., Desai J., Skinner I., Christie M., Hutchinson R. (2017). Examining the impact of regular aspirin use and PIK3CA mutations on survival in stage 2 colon cancer. Intern. Med. J..

[17] Reimers M.S., Bastiaannet E., Langley R.E., van Eijk R., van Vlierberghe R.L.P., Lemmens V.E.P., van Herk-Sukel M.P.P., van Wezel T., Fodde R., Kuppen P.J.K. (2014). Expression of HLA class I antigen, aspirin use, and survival after a diagnosis of colon cancer. JAMA Intern. Med..

[18] Wu W.K.K., Sung J.J.Y., Lee C.W., Yu J., Cho C.H. (2010). Cyclooxygenase-2 in tumorigenesis of gastrointestinal cancers: An update on the molecular mechanisms. Cancer Lett..

[19] Burn J., Gerdes A.-M., Macrae F., Mecklin J.-P., Moeslein G., Olschwang S., Eccles D., Evans D.G., Maher E.R., Bertario L. (2011). Long-term effect of aspirin on cancer risk in carriers of hereditary colorectal cancer: An analysis from the CAPP2 randomised controlled trial. Lancet.

[20] Tomozawa S., Nagawa H., Tsuno N., Hatano K., Osada T., Kitayama J., Sunami E., Nita M.E., Ishihara S., Yano H. (1999). Inhibition of haematogenous metastasis of colon cancer in mice by a selective COX-2 inhibitor, JTE-522. Br. J. Cancer.

[21] Yao M., Lam E.C., Kelly C.R., Zhou W., Wolfe M.M. (2004). Cyclooxygenase-2 selective inhibition with NS-398 suppresses proliferation and invasiveness and delays liver metastasis in colorectal cancer. Br. J. Cancer.

[22] Alfonso L., Ai G., Spitale R.C., Bhat G.J. (2014). Molecular targets of aspirin and cancer prevention. Br. J. Cancer.

[23] Chan A.T., Ogino S., Fuchs C.S. (2007). Aspirin and the risk of colorectal cancer in relation to the expression of COX-2. N. Engl. J. Med..

[24] Ferrández A., Piazuelo E., Castells A. (2012). Aspirin and the prevention of colorectal cancer. Best practice & research. Clin. Gastroenterol.

[25] Nosho K., Kawasaki T., Ohnishi M., Suemoto Y., Kirkner G.J., Zepf D., Yan L., Longtine J.A., Fuchs C.S., Ogino S. (2008). PIK3CA mutation in colorectal cancer: Relationship with genetic and epigenetic alterations. Neoplasia.

[26] Lee M.S., Menter D.G., Kopetz S. (2017). Right Versus Left Colon Cancer Biology: Integrating the Consensus Molecular Subtypes. J. Natl. Compr. Cancer Netw..

[27] Yamauchi M., Morikawa T., Kuchiba A., Imamura Y., Qian Z.R., Nishihara R., Liao X., Waldron L., Hoshida Y., Huttenhower C. (2012). Assessment of colorectal cancer molecular features along bowel subsites challenges the conception of distinct dichotomy of proximal versus distal colorectum. Gut.

[28] Gao M., Guo K.-M., Wei Y.-M., Ma M.-M., Cai J.-R., Xia T.-T., Ye Q.-J. (2017). Aspirin inhibits the proliferation of human uterine leiomyoma cells by downregulation of KRasp110α interaction. Oncol. Rep..

[29] Yu C., Li W.B., Liu J.B., Lu J.W., Feng J.F. (2018). Autophagy: Novel applications of nonsteroidal anti-inflammatory drugs for primary cancer. Cancer Med..

[30] Castoldi F., Humeau J., Martins I., Lachkar S., Loew D., Dingli F., Durand S., Enot D., Bossut N., Chery A. (2020). Autophagy-mediated metabolic effects of aspirin. Cell Death Discov..

[31] Huang Z., Fang W., Liu W., Wang L., Liu B., Liu S. (2018). Aspirin induces Beclin-1-dependent autophagy of human hepatocellular carcinoma cell. Eur. J. Pharmacol..

[32] Zeng X., Overmeyer J.H., Maltese W.A. (2006). Functional specificity of the mammalian Beclin-Vps34 PI 3-kinase complex in macroautophagy versus endocytosis and lysosomal enzyme trafficking. J. Cell Sci..

[33] Chen Z., Li Y., Zhang C., Yi H., Wu C., Wang J., Liu Y., Tan J., Wen J. (2013). Downregulation of Beclin 1 and impairment of autophagy in a small population of colorectal cancer. Dig. Dis. Sci..

[34] Robert Koch Institut (2016). Bericht zum Krebsgeschehen in Deutschland 2016.

[35] Barault L., Veyrie N., Jooste V., Lecorre D., Chapusot C., Ferraz J.-M., Lièvre A., Cortet M., Bouvier A.-M., Rat P. (2008). Mutations in the RAS-MAPK, PI(3)K (phosphatidylinositol-3-OH kinase) signaling network correlate with poor survival in a population-based series of colon cancers. Int. J. Cancer.

[36] Schmoll H.J., Stein A., Van Cutsem E., Price T., Hofheinz R.D., Nordlinger B., Daisne J.F., Janssens J., Brenner B., Reinel H. (2021). Pre- and Postoperative Capecitabine Without or With Oxaliplatin in Locally Advanced Rectal Cancer: PETACC 6 Trial by EORTC GITCG and ROG, AIO, AGITG, BGDO, and FFCD. J. Clin. Oncol..

